# *In vitro* Generation of Megakaryocytes and Platelets

**DOI:** 10.3389/fcell.2021.713434

**Published:** 2021-08-12

**Authors:** Huicong Liu, Jiaqing Liu, Lingna Wang, Fangfang Zhu

**Affiliations:** School of Biomedical Engineering, Shanghai Jiao Tong University, Shanghai, China

**Keywords:** platelet, megakaryocyte, hematopoietic stem cell, pluripotent stem cell, lineage reprogramming, transcription factor, histone deacetylase (HDAC), GABA

## Abstract

Platelets, the tiny anucleate cells responsible for stopping bleeding through thrombosis, are derived from hematopoietic stem cells through a series of differentiation steps. Thrombocytopenia, characterized by abnormally low blood platelet counts, may arise from cancer therapies, trauma, sepsis, as well as blood disorders, and could become a life-threatening problem. Platelet transfusion is the most effective strategy to treat thrombocytopenia, however, the source of platelets is in great shortage. Therefore, *in vitro* generation of platelets has become an important topic and numerous attempts have been made toward generating platelets from different types of cells, including hematopoietic stem cells, pluripotent stem cells, fibroblast cells, and adipose-derived cells. In this review, we will detail the efforts made to produce, in the *in vitro* culture, platelets from these different cell types. Importantly, as transfusion medicine requires a huge number of platelets, we will highlight some studies on producing platelets on a large scale. Although new methods of gene manipulation, new culture conditions, new cytokines and chemical compounds have been introduced in platelet generation research since the first study of hematopoietic stem cell-derived platelets nearly 30 years ago, limited success has been achieved in obtaining truly mature and functional platelets *in vitro*, indicating the studies of platelets fall behind those of other blood cell types. This is possibly because megakaryocytes, which produce platelets, are very rare in blood and marrow. We have previously developed a platform to identify new extrinsic and intronic regulators for megakaryocytic lineage development, and in this review, we will also cover our effort on that. In summary, stem cell-based differentiation is a promising way of generating large-scale platelets to meet clinical needs, and continuous study of the cellular development of platelets will greatly facilitate this.

## Introduction

Platelets, small and anucleate cells in the blood, are multifunctional and implicated in many pathophysiological processes, including hemostasis, thrombosis, vessel constriction and repair, and inflammations during host defense and tumor growth/metastasis (Harrison, 2005). Platelets, generated from hematopoietic stem cell-derived megakaryocytes, have a short lifespan of only 8–10 days (McArthur et al., 2018), and therefore in healthy persons, new platelets have to be produced constantly to maintain a normal level. Thrombocytopenia, defined by a platelet count of <1.5 × 10^11^/L in the blood, is not only commonly seen in some hematological diseases, for example, leukemia, bone marrow abnormalities, and hematopoietic aplastic anemia (Squires, 2015), but also may arise from multiple other conditions including connective tissue diseases, critical care medicine, hepatopathy, infectious illnesses, as well as cancer radiotherapy and chemotherapy (Robb and Begley, 1997). To reduce the mortality caused by bleeding in these situations, platelet transfusion has become an effective and irreplaceable treatment strategy.

**FIGURE 1:**
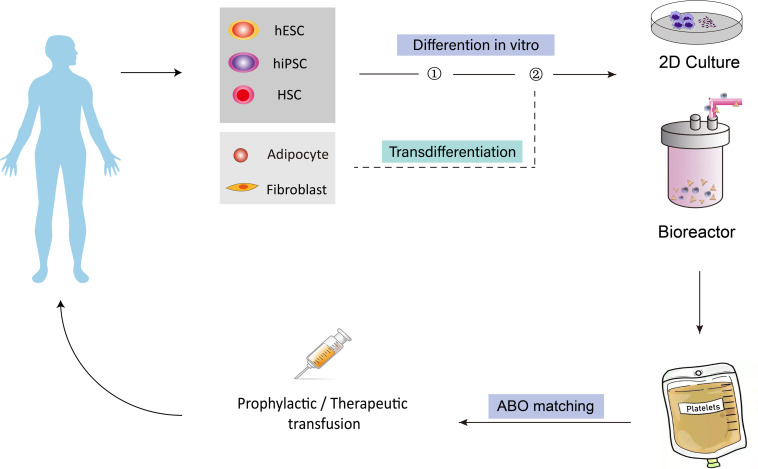
Overview of the concept of generating human platelets *in vitro* for clinical applications. Different cell types, including HSCs, hESCs, hiPSCs, fibroblasts and adipocyte-derived cells, could be induced to differentiate into MkPs/MKs, then bioreactors could be introduced to produce platelet from MK on a larger scale for transfusion purpose. The generated platelets could finally be injected into human for platelet-related diseases. HSCs, hematopoietic stem cells; hESCs, human embryonic stem cells; hiPSCs, human induced pluripotent stem cells; MkPs, megakaryocyte progenitors; MKs, megakaryocytes.

Currently, platelets used in the clinic are provided solely through blood donations. With the rising population of hematological cancer patients and the development of clinical treatment options for various diseases, demands for platelets in transfusion medicine have been steadily growing in the aging society. However, blood donations have not been increased proportionally, therefore, the severe shortage of platelets has become a worldwide problem. To address this issue, researchers have been focusing on pursuing alternative strategies to obtain platelets. Since the first report of *in vitro* generation of platelets from hematopoietic stem cells by Choi et al. (1995) about 30 years ago, efforts have been made to derive, from *in vitro* culture, human platelets from different types of cells, including hematopoietic stem cells, human embryonic stem cells, human induced pluripotent stem cells, fibroblast cells, and adipose tissue-derived cells. Among all these sources, stem cells have attracted the most attention because they secure an unlimited supply. To date, existing protocols have been established by the ectopic expression of key transcription factors (TFs) which control megakaryocyte and platelet cell fate during development, by the activation or inhibition of external signals with cytokines or chemical compounds, as well as by the co-culture with stromal cells or in 3D conditions to provide an environment similar to that during embryonic development. The ability to regenerate platelets *in vitro* would address the urgent and unmet needs of platelet supply in clinics and provide a promising way to solve the life-threatening bleeding problem in different diseases. Besides, these methods also have significant advantages over the current donor-dependent program, in terms of variations between donors, number of cells that could be obtained, risk of bacteria and virus contamination, cell viability, and storage, etc. Figure 1 shows how different cell sources could be used *in vitro* for platelet-required clinical applications.

However, only limited success has been achieved so far in producing truly mature and functional platelets from these *in vitro* cultures. With various differentiation methods, platelets could be generated, and they are phenotypically similar to plasma-derived platelets, however, the *in vivo* functions of these platelets were either not tested or not as good as plasma-derived platelets. More importantly, their lifespan is much shorter than that of primary platelets. Therefore, platelet differentiation protocols require further optimization. One of the possible reasons why *in vitro* derived platelets are not as expected is our lack of knowledge on how megakaryocyte and platelet generation from hematopoietic stem cells is precisely regulated during development. Megakaryocytes in the blood are very rare, and in bone marrow, they represent only 0.01% of all nucleated cells (Nakeff and Maat, 1974), which makes it difficult to isolate and culture these cells, and therefore, the research on megakaryocyte generation and differentiation falls far behind that of other hematopoietic cell types. We have described the identification of new intrinsic and extrinsic regulators for megakaryocyte development with an established platform that is designed to identify the hematopoietic regulatory network by combining the Gene Expression Commons (GeXC), which profiles the absolute expression of any gene on the microarray (Seita et al., 2012), CRISPR/Cas9 mediated gene knockout screening and lentivirus-mediated gene overexpression.

In this review, we will first summarize the existing research progress on *in vitro* generation of platelets from stem cells and other cell types and then discuss the promising solutions to hit the final goal of generating large-scale mature platelets *in vitro* for basic research and clinical applications.

## Cell Sources for *in vitro* Megakaryocyte and Platelet Generation

Different cells have been used as the starting materials to derive megakaryocytes and platelets *in vitro*, and these cells belong to two types: embryonic or adult stem cells including hematopoietic stem cells and human pluripotent stem cells which can directly differentiate into megakaryocytes and platelets naturally, while the other cell types including fibroblasts and adipose tissue-derived cells that are not its natural progenitors and require a conversion of cell fates from one to another. Stem cells as the cell source for platelets usually have the advantages in terms of scalability, ease of genetic modification, and platelet functions. However, different stem cells have their unique characteristics, and may require different methods to be directed to generate platelets. Table 1 summarizes current progress in platelet production *in vitro* from these different types of cells, which covers differentiation methods, efficiency, etc., and although all groups successfully characterized the generated megakaryocytes and platelets with various *in vitro* assays, functional tests in animal models (mouse, rabbit, etc.) were performed in only one third of these studies. Of note, Guan et al. (2017) not only demonstrated generated human megakaryocytes could engraft in mice and produce functional platelets, but also showed platelet recovery in non-human primates that were transplanted with autologous megakaryocytes, bringing one step closer to the clinical applications of human platelet transfusion.

**TABLE 1:** Summary of current methods for *in vitro* generation of megakaryocytes and platelets from different type of cells.

Reference	Cell source	Culture condition	Differentiation steps	Cytokines	Stromal cell coculture	Gene editing	Large scale	Efficiency	*In vivo* function evaluation
Choi et al., 1995	CD34+ HSC	2D	HSC-MK-PLT	N/A	N/A	N/A	No	2.1 MKs per HSC	N/A
Norol et al., 1998		2D	HSC-MkP-MK-PLT	TPO, SCF, IL-3, IL-6	N/A	N/A	No	4.6 MKs per HSC	N/A
Proulx et al., 2004		2D	HSC-MK-PLT	TPO, SCF, IL-6, FL	N/A	N/A	No	120 PLTs per seeded cells	N/A
Matsunaga et al., 2006		2D	HSC-MK-PLT	TPO, SCF, IL-3, FL, IL-6,IL-11, IL-1β, SDF-1α, FGF-4, PDGF	hTERT	N/A	Yes	3.36 × 10^4^ PLTs per HSC	N/A
Yang et al., 2016		Rotary vessel	HSC-MK-PLT	TPO, SCF, IL-3, IL-11	N/A	N/A	Yes	∼1.9 × 10^3^ PLTs per HSC	N/A
Guan et al., 2017		2D	HSC-MK	SCF, FL, TPO, IL-3, IL-6, IL-11, GM-CSF, SR1	N/A	N/A	No	1 × 10^4^ MKs per HSC	MKs produced functional PLTs in mice and monkeys
Guan et al., 2020		Roller-bottle	HSC-MK	TPO, SCF, IL-6, SR1, C433, VPA	N/A	N/A	Yes	2 × 10^4^ MKs per HSC	MKs produced functional PLTs in mice
Gaur et al., 2006	hESC	2D	hESC-MK-PLT	TPO, bFGF	OP9, MEF	N/A	No	0.05–0.2 MK per hESC	N/A
Takayama et al., 2008		2D	hESC-HPC-MK-PLT	TPO, IGF-II, VEGF, bFGF, heparin, SCF, PlGF	OP9, C3H10T1/2	N/A	No	48 PLTs per hESC	N/A
Lu et al., 2011		2D	hESC-MK-PLT	IL-6, IL-9, IL-11, bFGF, VEGF, TPO, SCF	OP9, C3H10T1/2	N/A	Yes	5.5 × 10^3^ PLTs per hESC	PLTs contributed to thrombi in mice
Pick et al., 2013		2D	hESC-MkP-MK-PLT	TPO, SCF, IL-3, FGF2, VEGF, BMP-4	MEF	N/A	No	0.25–1.6 MkPs per 10^3^ hESCs	N/A
Takayama et al., 2010	hiPSC	2D	hiPSC-HPC-MK-PLT	SCF, TPO, FL	C3H10T1/2	c-MYC	No	20 PLTs per hiPSC	PLTs contributed to thrombi in mice
Nakamura et al., 2014		2D	hiPSC-HPC-imMKCL-MK-PLT	TPO, SCF	C3H10T1/2	c-MYC, BMI1, BCL-XL	No	250 MKs per imMKCL	PLTs contributed to thrombi in mice
Ito et al., 2018		Bioreactor	hiPSC-imMKCL-MK-PLT	SCF, TA-316, Y27632/Y39983, SR1, KP-457, GNF-351	C3H10T1/2	c-MYC, BMI1, BCL-XL	Yes	70–80 PLTs per hiPSC	PLTs contributed to hemostasis in mice and rabbits
Feng et al., 2014		2D	hiPSC-MkP-MK-PLT	TPO, SCF, IL-3, IL-6, IL-9, ACF, FL, APEL, BMP-4, VEGF, bFGF	N/A	B2M KO	No	∼16 MkPs per iPSC	PLTs contributed to thrombi in mice
Moreau et al., 2016		2D	hiPSC-hPSC-MK	TPO, IL-1β, SCF, BMP4, Y-27632, FGF2, LY-294002	N/A	GATA1, FLI1, TAL1	Yes	2 × 10^5^ MKs per hiPSC	N/A
Eicke et al., 2018		Bioreactor	hiPSC-MK-PLT	VEGF, BMP4, TPO, SCF, IL-3, Y27632	N/A	N/A	Yes	29.9 MKs per hiPSC	MKs produced PLTs in mice
Norbnop et al., 2020		2D	hiPSC-HPC-MK-PLT	VEGF, TPO, SCF, heparin	OP9	B2M KO	No	N/A	MKs produced PLTs in mice
Ono et al., 2012	Fibroblast	2D	Fibroblast-iMK-PLT	N/A	N/A	p45NF-E2, Maf G, MafK, CEBPα	No	0.04–0.05 iMK per fibroblast	MKs produced PLTs in mice
Pulecio et al., 2016		2D	Fibroblast-iMkP-MK-PLTs	TPO, IL-3, IL-6, IL-9, SCF	N/A	GATA1, GATA2, TAL1, LMO2, c-MYC, RUNX1	No	N/A	MkP produced MKs and PLTs in mice
Matsubara et al., 2009	Adipocyte	2D	Adipocyte-MK-PLT	LDL, TPO, dNTP, Insulin, Transferrin	N/A	N/A	No	0.2 MK and 0.015 PLT per adipocyte precursor cell	N/A
Tozawa et al., 2019		Bioreactor	Adipose-ASCL-MK-PLT	Transferrin, LDL, Insulin, dNTP, TPO	N/A	N/A	Yes	0.42 PLT per ASCL	PLTs survived in mice

*HSC, hematopoietic stem cell; hESC, human embryonic stem cells; hiPSC, human induced pluripotent stem cell; MkP, megakaryocyte progenitor; MK, megakaryocyte; PLT, platelet.*

### Generation of Platelets From Hematopoietic Stem Cells

Hematopoietic stem cells (HSCs), which could be found in bone marrow (BM), peripheral blood (PB), and cord blood (CB) after birth, continuously divide to provide more hematopoietic stem and progenitor cells to balance self-renewal and differentiation. Through multiple steps, HSCs differentiate into multipotent progenitors (MPPs), common myeloid progenitors (CMPs), megakaryocyte-erythroid progenitors (MEPs), megakaryocyte progenitors (MkPs), and then megakaryocytes (MKs), which finally mature and produce functional platelets (Seita and Weissman, 2010; Machlus and Italiano, 2013). Therefore, the generation of HSC-derived platelets seems to be a natural process, and the earliest attempts to create platelets *in vitro* were using CD34-enriched HSCs as the starting materials. Choi et al. (1995) first described a method in which isolated HSCs from PB were cultured in conditions supplemented with human and aplastic canine serum to induce MK differentiation. Aplastic canine serum was then removed from the culture medium in which plasma factors from human serum promoted MK differentiation to platelets, which exhibited a similar ultrastructure to platelets isolated from plasma and could be aggregated after platelet agonist adenosine diphosphate (ADP) treatment. However, the differentiation efficiency is very low, with only 3 × 10^6^ mature MKs obtained from one leukapheresis unit, and only about 40% MKs could generate platelets upon further differentiation (Choi et al., 1995).

Various optimizations were later tried to improve the differentiation efficiency. Norol et al. (1998) searched for cytokines that could stimulate the generation of MKs and platelets from HSCs. They tested the functions of megakaryocyte growth and development factor (MGDF, now commonly known as thrombopoietin, TPO), the ligand for Mpl, either alone or in various combinations with stem cell factor (SCF), interleukin-3 (IL-3), and IL-6. They found both TPO alone and its combination with these three cytokines accelerated MK differentiation, while TPO alone was able to promote platelet production by 10-fold, highlighting the critical role of TPO in MK differentiation, maturation, and platelet generation (Norol et al., 1998). Currently, TPO is regarded as one of the most important cytokines in megakaryocytopoiesis and thrombopoiesis. Proulx et al. (2004), in an interesting study, showed the unexpected effect of elevated temperature on differentiation of HSCs to MK-committed cells. Compared to those at 37°C, the differentiation cultures maintained at 39°C promoted the proliferation and differentiation efficiency of HSCs, accelerated MK maturation by 3–4 days, and improved platelets output by more than 15-fold (Proulx et al., 2004). Later, the 3D culture method, specifically the roller-bottle cell culture system, was also found to be able to improve the efficiency of platelet generation from CB HSCs by a few folds, compared with the routinely used static culture condition, either in a small research scale (Yang et al., 2016) or in a Good Manufacturing Practice (GMP) standard culture (Guan et al., 2020).

To fulfill transfusion purposes, attempts were also made to generate platelets on a large scale. Matsunaga et al. (2006) reported a 33-day three-phase culture system using HSCs isolated from CB. By combining co-culture with telomerase gene-transduced human stromal cells for MK differentiation and expansion, and liquid culture medium for platelet production, they were able to obtain 1.26–1.68 × 10^11^ platelets from 1 unit of CB (which contains 3–5 × 10^6^ CD34^+^ HSCs). These platelets exhibited features similar to those from plasma in both morphology and *in vitro* functions, including ADP-induced aggregation (Matsunaga et al., 2006).

However, despite all the current progress, the biggest challenge of producing platelets from HSCs is still the low differentiation efficiency and the difficulty of large-scale production in a time- and cost-efficient manner. Besides, there is no detailed analysis of the production of each progenitor population (CMPs, EPs, and MkPs) during the differentiation process, which led to the incomplete establishment of the differentiation path from HSCs to platelets.

### Generation of Platelets From Human Pluripotent Stem Cells

Human pluripotent stem cells (hPSCs) comprise human embryonic stem cells (hESCs) and the recently discovered human induced pluripotent stem cells (hiPSCs). hPSCs could proliferate (self-renew) infinitely *in vitro*, and under specific conditions, they can differentiate into any cell type of the human body. Therefore, hPSCs could serve as an unlimited source for platelet production *in vitro*.

#### Generation of hESC-Derived Platelets

Thomson et al. (1998) reported the derivation of the first hESCs from the inner cell mass of blastocyst-stage human embryos, opening the door for platelet differentiation from hESCs. Gaur et al. (2006) established an OP9 stromal cell co-culture system to increase MK production from hESCs, while the yield was unsatisfactory (less than 1 MK produced per 10 input hESCs) and no release of platelets was demonstrated. Takayama et al. (2008) further refined the scheme, in which hESCs were co-cultured with either C3H10T1/2 or OP9 stromal cells and supplemented with human vascular endothelial growth factor (VEGF) to promote the emergence of sac-lick hematopoietic progenitors, which were then further differentiated into MKs and platelets by adding TPO. The platelets displayed activation in response to ADP. This study showed that adding factors that promote mesoderm differentiation from hESCs will eventually benefit the MK and platelet generation. However, MKs generated in this system produced very few platelets (Takayama et al., 2008). Later, Lu et al. (2011) described a more competent method to obtain functional MKs and platelets from hESCs on a large scale, in which they isolated hESC-derived early hematopoietic progenitor cells, the hemangioblasts/blast cells, for further differentiation into MK lineage cells through coculture with OP9. They were able to generate over 100 CD41a^+^ MKs per hESC, an efficiency much higher than the previous 0.05–0.2 MKs and 2–5 MKs as reported by Gaur et al. (2006) and Takayama et al. (2008), respectively. After being transplanted into a mouse model, these platelets form thrombi at the sites of injury, which is the first report demonstrating the *in vivo* function of platelets derived from hESCs. However, only fewer than 7 platelets were produced from each hESC-derived MK with this method (Lu et al., 2011). A serum- and feeder-free culture system through embryoid bodies (EBs) differentiation was also established, moving one step forward toward using hESC-derived platelets for transfusion medicine, though the differentiation efficiency is very low, with only 100–800 MK-containing cell colonies obtained per 100,000 sorted CD41a+ cells derived from hESCs (Pick et al., 2013).

These studies above suggest that hESC-derived platelet production is feasible.

#### Generation of hiPSC-Derived Platelets

Human induced pluripotent stem cells possess similar self-renewal capacity and multipotency to hESCs and could be generated from somatic cells of any individual by overexpression of the TF combinations OCT3/4, SOX2, KLF4, and c-MYC (OSKM) or OCT3/4, SOX2, NANOG, and LIN28 (OSNL) (Takahashi et al., 2007; Yu et al., 2007), which is a breakthrough in the stem cell field. Therefore, hiPSCs represent another promising unlimited source to obtain platelets without the risk of immune rejection, particularly for patients with a rare HLA, and ethical concerns of embryo destruction related to hESCs. Takayama et al. (2010) first demonstrated OSKM hiPSCs could give rise to CD41a^+^CD42b^+^ platelets. They co-cultured hiPSCs with mouse cell line C3H10T1/2 for differentiation into hematopoietic progenitors, which were subsequently cultured in MK differentiation medium to produce platelets. *In vivo* imaging revealed that these CD42b^+^ platelets were present in thrombi after laser-induced vessel wall injury. Importantly, they showed that c-MYC promoted megakaryopoiesis in the early stage of differentiation but later inhibited thrombopoiesis, indicating complicated roles c-MYC is playing in this differentiation process (Takayama et al., 2010). Nakamura et al. (2014) developed inducible imMKCLs cell lines to improve the consistency and efficiency of platelet generation from hiPSCs. The imMKCLs cell lines are immortalized megakaryocytic cell lines which were differentiated from hiPSCs by inducible overexpression of c-MYC, BMI1, and BCL-XL through the Tet-on system, and then the expression of these three genes would be shut down when further differentiation of imMKCLs into platelets is needed. The imMKCLs could be expanded *in vitro* for 4–5 months, after which they could still be differentiated into platelets that are similar to those isolated from blood (Nakamura et al., 2014). In 2018, turbulence, which mimics the shear stress of blood and helps cut megakaryocytes into small platelets, was introduced by Ito et al. (2018) on a large-scale generation of platelets from hiPSCs. They established a 3D differentiation system in which each hiPSC-derived MK generated 70–80 platelets, almost a 20-fold increase compared with previous reports, although it is still much less than that of 10^4^ platelets per megakaryocyte in the human body (Machlus and Italiano, 2013). Nevertheless, they eventually collected 100 billion platelets from hiPSC-MKs in an 8L turbulence-controllable bioreactor, which represents a breakthrough for *in vitro* generation of platelet on a clinical scale for transfusion medicine (Ito et al., 2018).

Besides, other methods were also developed to induce the generation of platelets from hiPSCs. These methods either utilize stepwise differentiation from hiPSCs to early hematopoietic progenitors, then to MKs and finally to platelets, by using different cytokine combinations and culture media (Feng et al., 2014), or applied the microcarrier beads assisted stirred bioreactors to promote MK generation (Eicke et al., 2018), or rely on ectopic expression of key MK TFs GATA1, FLI1, and TAL1 in hiPSCs to directly promote platelet generation (Moreau et al., 2016). 2 × 10^11^ MKs releasing 1 × 10^12^ platelets – the equivalent of 3 transfusion units – could be obtained from only 1 × 10^6^ input hiPSCs (Moreau et al., 2016). Furthermore, by knocking out the β2-microglobulin gene, several groups have generated platelets without major histocompatibility antigens (HLA), which theoretically does not require additional HLA-match and does not cause immune rejection in clinical applications (Feng et al., 2014; Norbnop et al., 2020; Suzuki et al., 2020). Therefore, the application of hiPSC-based technology could potentially yield a consistent supply of HLA- and/or HPA-matched or even autologous platelets for clinical applications.

### Generation of Platelets From Other Cell Types

Besides all studies described above to use various stem cells as the starting materials for platelet *in vitro* production, other cell types have also been used to generate megakaryocytes and platelets. However, the efficiency is low, and it is still far away from a practically feasible clinical implementation for platelet transfusion. Alternatively, they can be used for *in vitro* disease modeling and drug screening purposes for platelet-related disorders.

#### Generation of Fibroblast-Derived Platelets

Transdifferentiation, also known as lineage reprogramming, was recognized as the direct conversion from one mature cell type to another. After the generation of hiPSCs from fibroblasts by overexpression of OSKM or OSNL, fibroblasts or other mature cells have been successfully converted to many different types of cells, such as neurons, cardiomyocytes, hepatocytes, etc. (Wang et al., 2021). Therefore, researchers have also attempted and accomplished, through overexpression of TFs critical for megakaryocytic lineage development, the direct conversion of fibroblasts to MKs, which could further produce platelets *in vitro* or *in vivo* after transplantation into mice. In one study, 3T3 fibroblast cell line and adult human dermal fibroblasts could be transdifferentiated into induced megakaryocytes (iMKs) through the overexpression of p45NF-E2 and its binding proteins MafG and MafK (Ono et al., 2012). iMKs could further produce CD41^+^ platelets which were shown to be involved in thrombosis on the collagen-coated chip *in vitro*. However, the conversion efficiency is pretty low, and they only obtained 8–10 × 10^5^ iMKs from 20 × 10^6^ fibroblasts, indicating further optimization is required. Pulecio et al. (2016), in a previous study, identified a set of TFs GATA1, TAL-1, LMO2, and c-MYC (GTLM) that was able to convert fibroblast cells into EPs in the presence of erythropoietin (EPO). As EPs and MkPs are both derived from MEPs during development, they may share some core transcriptional regulators. Indeed, in a later study, they successfully generated MkPs from human fibroblasts by adding TFs GATA2 and RUNX1 to the GTLM combo (Pulecio et al., 2016). These MkPs could further differentiate and produce platelets *in vitro* as well as generate CD41^+^/CD42^+^ platelets *in vivo* after transplantation into immunocompromised mice. This direct conversion to MkPs takes only 12 days and the CD41^+^/CD45^+^ cells appear in 8% cell population. Importantly, they also showed in a Fanconi Anemia patient which has extremely low HSCs and frequent thrombocytopenia, that fibroblast cells could be isolated and *in vitro* gene-corrected, and then directly converted to generate healthy megakaryocytes and platelets.

#### Generation of Adipose Tissue Cell-Derived Platelets

Matsubara et al. (2009) have been trying to study the capacity of adipose tissue-derived cells in differentiating into MKs and platelets. In 2009, they first showed that megakaryocytes and platelets could be generated from human subcutaneous adipose tissues (Matsubara et al., 2009). Later, to further increase the differentiation efficiency, they generated an adipose tissue-derived mesenchymal stem cell line (ASCL) from adipose-derived stromal cells. As the starting materials, these ASCL cells were able to proliferate for more than 2 months *in vitro*, and upon differentiation, these cells could generate platelets, which express typical platelet cell surface markers and could be activated by ADP. In the end, they were able to obtain one platelet from one starting ASCL (Tozawa et al., 2019).

## Regulatory Network of Megakaryocyte Development

Despite all current progress, the generation of truly mature and functional platelets from *in vitro* cultures was demonstrated in few studies, and one possible reason is, the percentage of MKs in bone marrow is very low (about 0.01% of all nucleated cells) (Nakeff and Maat, 1974), therefore, although MKs were discovered over 100 years ago, it is very difficult to study their development and biology. So far, researchers have been focusing on the study of internal and external factors that affect the fate of megakaryocytic lineage cells. The external factors are exogenous signal transduction and microenvironment, and internal factors are mainly TFs and epigenetic regulators. To date, only a few TFs have been reported to be involved in this process, including GATA1 (Crispino and Horwitz, 2017), FLI1 (Li et al., 2015), MEIS1 (Zeddies et al., 2014), RUNX1 (Goldfarb, 2009), and SCL (Robb and Begley, 1997). Not surprisingly, some of these genes, including RUNX1, ERG and MEIS1, and TPO-encoding gene Thrombopoietin have all been demonstrated to be involved in hESC *in vitro* differentiation into MKs and platelets (Tu et al., 2017; Wang et al., 2018; Zhang et al., 2018), indicating that studies in MK regulators will eventually help establish methods to drive platelet *in vitro* generation.

Our laboratory is devoted to discovering more TFs and mapping the regulatory network to understand the decision-making factors during platelet development (Zhu et al., 2018). To identify the regulators for HSC differentiation to MkPs, the immediate precursor cells for MKs and platelets, we applied Gene Expression Commons (GeXC), a platform developed in Irving Weissman’s laboratory at Stanford University (Seita et al., 2012), to get the candidate gene list, in which these genes have high expression level in MkPs but low in EPs and MEPs, a common progenitor for MkPs and EPs. This expression pattern indicates that genes in this list may play a role in MkP differentiation. To address this hypothesis, we deleted these candidate genes separately in HSCs using CRISPR/Cas9 and found that the knockout of some genes inhibited MkP generation from HSCs. Subsequently, 9 candidate TFs, which include MZF1, GSX2, HOXC6, HES7, FOXB1, MXD3, HOXA9, NFATC1, and PCGF2, were identified to promote MkP generation from HSCs by lentivirus-mediated overexpression. Further analysis showed that MkP generation was increased by four–five fold with this TFs overexpression. To our knowledge, the functions of these 9 TFs in MK lineages development have not been reported previously. We also found inhibition of histone deacetylase (HDAC) activity could also promote the differentiation of HSCs into MkPs and platelets, possibly through regulating some of the 9 newly identified TFs: gene expression analysis showed GSX2, MXD3, HOXC6, and HES7 were significantly up-regulated while PCGF2, FOXB1, and MZF1 were moderately up-regulated after HDAC inhibition. These results have added more players into the regulatory network of endogenous factors during MkP development and platelet generation.

In another study to identify the exogenous signals, we found the neurotransmitter GABA was involved in the occurrence of platelets, indicating there is a link between the neural and hematopoietic systems (Zhu et al., 2019). We found GABRR1 is the only GABA receptor that is expressed in subsets of HSCs and MkPs. Electrophysiological recording experiment showed that GABRR1^+^ but not GABRR1^–^ HSCs and MkPs respond to GABA in patch-clamp studies. Besides, GABA signaling through GABRR1 affected mouse HSC differentiating into megakaryocytic lineage cells. Stimulated by GABA agonists, including TACA and Muscimol, the mouse showed increased platelet numbers. We also found similar functions of GABRR1 in human hematopoiesis. Treatment with GABRR1 antagonist inhibited the number of MkPs and platelets generated from HSCs, while agonist treatment or overexpression of GABRR1 in HSCs significantly promoted MkP and platelet generation. Thus, this is the first study that showed the role of GABA signaling in the differentiation of HSCs and MkPs, and provides another evidence for the correlation between the neural and hematopoietic systems. This study not only indicates GABA may be a potential target in platelet-related disorders, but also shows it may help increase the MK and platelet production from HSCs (and potentially other stem cells) by adding GABA or its agonists during *in vitro* differentiation cultures.

Taken together, our studies reveal the complexity and the lack of knowledge of comprehensive MK regulators. Our laboratory is devoted to identifying more MK lineage-specific genes and signaling factors and completing the regulatory network of platelet generation, which may hopefully not only provide new drug targets related to platelet counts, activation, and aggregation, but also facilitate platelet generation *in vitro*.

## Discussion

Currently, various methods have been developed and great progress has been made toward the generation of platelets from different cell types *in vitro* to fulfill the transfusion medicine purpose independent of or as a supplement to blood donations. However, certain limits still exist in using each of these cell types as the starting materials for platelet *in vitro* biogenesis, and these problems and/or questions must be resolved before they could be used in clinical applications.

Due to the rareness of HSCs in PB and the difficulty in obtaining BM, HSCs from CB are now routinely used for *in vitro* platelet differentiation. Based on this, new cytokines (such as IL-11) and new chemical compounds (such as Y27632 and DAC) have been identified, a new 3D culture system (such as roller-bottle culture system), and new stromal cells (such as hTERT-transduced stroma) have been introduced to the culture conditions. These optimizations have significantly improved the differentiation efficacy, with 2 × 10^4^ MKs obtained from 1 seeded CD34^+^ HSC, compared with the previous 5 MKs per HSC (Norol et al., 1998; Guan et al., 2020), and 1.26 × 10^11^–1.68 × 10^11^ platelets generated from one CB unit, which equal to 2.5–3.4 units of donor-derived platelets (Matsunaga et al., 2006). However, only a limited number of CD34^+^ HSCs, about 3-5 × 10^6^, could be extracted from one unit of CB, and these cells are difficult to obtain and culture. Besides, the number of platelets generated by current differentiation methods still cannot meet the requirement of transfusion medicine, therefore, more work is needed to improve the efficacy for large-scale generation of platelets *in vitro*. It has also been reported that, although CB HSCs were able to yield the highest number of MKs compared with those from PB and BM, the CB-derived MKs showed reduced ploidization indicating less maturation (van den Oudenrijn et al., 2000). Last but not least, there is no detailed analysis of the production of each progenitor population during the differentiation process, which led to the incomplete establishment of the differentiation path from HSCs to platelets.

Human embryonic stem cells and hiPSCs can proliferate indefinitely *in vitro*, which may bypass some limits associated with CB-derived HSCs as the starting materials. However, hESCs are derived from blastocyst-stage embryos (Thomson et al., 1998), so there are ethical controversies and potential risks of viral infection and congenital diseases in the human embryos. hiPSCs are derived from human somatic cells (Takahashi et al., 2007; Yu et al., 2007), thus avoid ethical controversy as in hESCs, but there are still some obstacles to use hiPSCs for *in vitro* generation of platelets, which include safety issues, long differentiation cycles, and the huge cost. One safety concern is the residual undifferentiated hiPSCs in the final products, as hiPSCs have the potential to form tumors after transplantation *in vivo*. Considering that a large number of platelets is needed for each transfusion (about 3 × 10^11^ platelets per unit), even contamination of 0.01% hiPSCs in platelets means 3 × 10^7^ undifferentiated hiPSCs in a treatment unit, which is more than enough to form tumors in human. Although platelet products are usually irradiated by gamma-ray before transfusion, one still cannot exclude the possibility of hiPSC contamination after irradiation. Another safety issue is that the oncogene c-MYC was overexpressed for the best results of platelet generation (Takayama et al., 2010; Nakamura et al., 2014; Ito et al., 2018), which leads to the risk of oncogenicity. Even though regulated by Doxycycline, the c-MYC gene may be reactivated in other ways. As for the production cycle, it takes weeks or even months to establish the hiPSC cell lines and carry out various assays to identify the best clones (Takahashi et al., 2007; Yu et al., 2007). Then, the differentiation of hiPSCs into platelets involves multiple steps and a long period. For example, in a study by Ito et al. (2018), it takes about 14 days to differentiate hiPSCs into hematopoietic progenitors *in vitro*, then several weeks to establish and expand imMKCLs by overexpression of c-MYC, BMI1, and BCL-XL, finally, another 6 days are needed to obtain mature platelets from these expanded imMKCLs. In another study by Feng et al. (2014), it takes 6 days to direct hiPSCs *in vitro* to hematopoietic progenitors, after which early MkPs were generated in another 7 days, a final 7 days are then required to induce mature MKs and platelet generation. The cost associated with hPSC-derived platelets is mainly due to the use of growth factors/small molecules and plasma/serum during the cell culture medium. Some growth factors like bFGF are indispensable in the routine culture of hPSCs, and more cytokines are needed in the platelet differentiation from these hPSCs. Ito et al. (2018) introduced human plasma/serum, SCF, TA-316, KP-457SR-1, GNF-351, and Y27632 at multiple stages of their differentiation method, and Feng et al. (2014) used even more cytokines, including BMP-4, VEGF, bFGF, TPO, SCF, Flt3-L, IL-3, IL-6, and IL-9. A common issue with both hESC- and hiPSC-derived platelets is, these generated platelets have a much shorter lifespan than those from plasma (Feng et al., 2014; Moreau et al., 2016), indicating these differentiated platelets are not as fully mature and functional as primary platelets.

Transdifferentiation from fibroblasts to platelets is not feasible for making large-scale platelets due to the limited sources, difficulty in expansion of fibroblasts, as well as low conversion efficiency (Ono et al., 2012; Pulecio et al., 2016). As to using adipose tissue-derived cells for *in vitro* platelet production, although expandable cell lines could be established from adipocytes, it is difficult to use these cells in large-scale manufacture because of their limited proliferation and inefficient differentiation. Moreover, the final products not only express typical platelets cell surface markers but also possess some features of mesenchymal stem cells, such as expression of CD90, indicating that these final products are not bona fide platelets, and the mechanism of adipocytes converting to platelets is still unknown (Tozawa et al., 2019).

In summary, although significant progress has been made toward the *in vitro* generation of platelets from HSCs, hPSCs, fibroblasts, and adipose-derived cells, with various stem cells as the most promising starting materials, the current issues of scalability, cost, duration of differentiation, and cell functionality are all hurdles to applying these *in vitro* generated platelets into clinical applications. A better understanding of the biology and development of megakaryocytes will hopefully address some of these issues and greatly facilitate *in vitro* platelet biogenesis on a large scale for transfusion medicine.

## Author Contributions

FZ contributed to the conception and design of the structure of the manuscript and wrote the first draft of the manuscript. HL collected information from reference literature, organized the database, and wrote some sections of the manuscript. FZ, HL, JL, and LW contributed to manuscript revision and read and approved the submitted version of the manuscript. All authors contributed to the article and approved the submitted version.

## Conflict of Interest

The authors declare that the research was conducted in the absence of any commercial or financial relationships that could be construed as a potential conflict of interest.

## Publisher’s Note

All claims expressed in this article are solely those of the authors and do not necessarily represent those of their affiliated organizations, or those of the publisher, the editors and the reviewers. Any product that may be evaluated in this article, or claim that may be made by its manufacturer, is not guaranteed or endorsed by the publisher.
